# Extracellular Matrix and Endothelial Activation Markers in the Bronchial and Pulmonary Arteries in COPD

**DOI:** 10.1111/crj.70141

**Published:** 2025-11-29

**Authors:** Raquel Annoni, Jôse Mára de Brito, Salvatore Battaglia, Natália de Souza Xavier Costa, Ligia Braga Lopes Couceiro, Renata Calciolari Rossi, Marisa Dolhnikoff, Pieter S. Hiemstra, Klaus F. Rabe, Peter J. Sterk, Thais Mauad

**Affiliations:** ^1^ Laboratório de Patologia Ambiental e Experimental, Departamento de Patologia Faculdade de Medicina da Universidade de São Paulo São Paulo SP Brazil; ^2^ Graduate Program in Rehabilitation Sciences, School of Physical Education, Physical Therapy and Occupational Therapy Universidade Federal de Minas Gerais Minas Gerais Brazil; ^3^ Instituto Básico de Biociências, Universidade de Taubaté (UNITAU) Taubaté SP Brazil; ^4^ Division of Respiratory Diseases Department of Promozione della Salute, Materno Infantile Medicina Interna e Specialistica di Eccellenza 'G. D'Alessandro' – PROMISE – University of Palermo Palermo Italy; ^5^ Departamento de Patologia Faculdade de Medicina da Universidade do Oeste Paulista Presidente Prudente SP Brazil; ^6^ Department of Pulmonology Leiden University Medical Center Leiden The Netherlands; ^7^ LungenClinic Grosshansdorf, Member of the German Center for Lung Research (DZL) Grosshansdorf Germany; ^8^ Professor Emeritus University of Amsterdam Amsterdam The Netherlands

**Keywords:** bronchial artery, chronic obstructive pulmonary disease, endothelial activation, extracellular matrix, fibronectin, pulmonary artery, vascular remodelling

## Abstract

**Background:**

Vascular alterations contribute significantly to the chronic obstructive pulmonary disease (COPD) pathophysiology. Thirty‐nine percent of patients with COPD develop pulmonary hypertension, especially patients with the severe form of the disease. Cigarette‐induced endothelial dysfunction is important in the pathogenesis of vascular alterations, even in the mild forms of the disease. This study investigates extracellular matrix (ECM) remodelling and endothelial activation in pulmonary (PMA) and bronchial muscular arteries (BMA) from non‐smokers (NS), nonobstructed smokers (NOS) and patients with mild/moderate COPD.

**Methods:**

Lung tissue samples from 44 patients undergoing lung resection were analysed. Morphometric parameters, ECM components (collagens, fibronectin, elastic fibres, tenascin‐C and versican) and endothelial markers (VCAM‐1, ICAM‐1 and endothelin‐1) were quantified in BMA and PMA using immunohistochemistry and morphometric analysis. Group differences and correlations with clinical parameters were assessed.

**Results:**

COPD patients showed increased intimal thickness and fibronectin deposition in PMA, and larger adventitial areas in BMA compared to NS. NOS exhibited higher VCAM‐1 expression in BMA and increased elastic fibre content in PMA. In COPD, elastic fibres and type‐III collagen negatively correlated with smoking history (pack‐years), while fibronectin positively correlated with age. VCAM‐1 expression in BMA correlated negatively with lung function (FEV_1_ and FEV_1_/FVC).

**Conclusions:**

This study demonstrates for the first time ECM remodelling and endothelial activation in bronchial arteries of smokers and patients with COPD. Fibronectin emerges as a key ECM component in arterial remodelling in mild–moderate COPD. Understanding vascular changes may provide new insights into the regulation of bronchial circulation and the development of pulmonary hypertension in COPD.

## Graphical abstract

- We investigated extracellular matrix remodelling and endothelial activation in both pulmonary and bronchial arteries in smokers and patients with mild/moderate COPD.
- There was increased expression of the adhesion molecule VCAM‐1 in the bronchial artery of smokers.
- In COPD, intimal thickening of the pulmonary artery was associated with increased expression of the inflammation related extracellular matrix fibronectin.

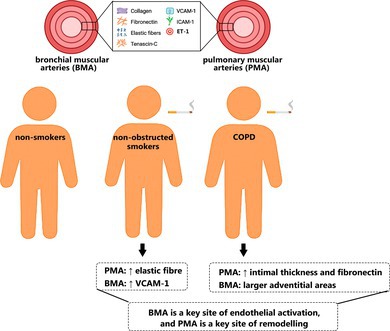

## 1 Introduction

Chronic obstructive pulmonary disease (COPD) is characterized by persistent respiratory symptoms and airflow limitation due to abnormalities of the airway, alveolar and blood vessels. Such abnormalities are associated with extracellular matrix (ECM) remodelling, characterized by tissue destruction and abnormal ECM deposition [1]. Much less studied than airways and alveoli [2], alterations in larger pulmonary vessels represent an important component of the disease, since these abnormalities may further impair gas exchange and lead to pulmonary hypertension (PH) [3]. It is estimated that 39% of patients with COPD, particularly those with the severe form, develop pulmonary hypertension [4]. It has been suggested that vascular pathology may develop early in cigarette smoke‐induced respiratory diseases.

Indeed, endothelial changes and vessel remodelling such as smooth muscle cell proliferation and elastin/collagen deposition in the thickened intima of pulmonary arteries occur in smokers and mild cases of COPD [5]. Several mechanisms are believed to cause vessel abnormalities in smoking and COPD. Cigarette smoke induces loss of nitric oxide (NO) and increased reactive oxygen species (ROS), causing endothelial dysfunction and vasoconstriction, with endothelin‐1 (ET‐1) being a mediator of this process [6]. Increased circulating concentrations of adhesion molecules have been identified in COPD patients [7], contributing to endothelial dysfunction. In COPD patients, chronic hypoxia, loss of the capillary bed by alveolar destruction and systemic inflammation contribute to further pulmonary vascular alterations eventually leading to pulmonary hypertension [8].

The lungs are supplied by two different vascular systems consisting of the pulmonary and bronchial arteries. There is scarce information on the bronchial artery of smokers and patients with COPD that may also be affected by smoking and chronic hypoxia. The bronchial arteries provide nourishment to the supporting structures of the lungs and do not participate in gas exchange [9]. The bronchial circulation responds to chronic pulmonary ischaemia and decreased pulmonary blood flow with hypertrophy or enlargement in an effort to maintain blood flow to the affected lung. A functional bronchial circulation is required for the maintenance of normal mucociliary transport, since it is important in the formation of the epithelial lining fluid which plays a role in the local defences against inhaled irritants and substances [10].

Only a single study performed more than 50 years ago addressed the bronchial circulation in 15 patients with COPD by selective catheterization and roentgenographic visualization, showing enlargement of the bronchial artery in all patients, but without correlation with pulmonary vessel size, pulmonary function and clinical symptoms [11]. More recent studies addressed the bronchial microcirculation only, at capillary level, via endobronchial biopsies in COPD, showing no alterations in some studies, while in others, an enlarged microcirculation was detected [12].

The ECM is a 3D extracellular network that comprises collagens, elastic fibres, glycosaminoglycan chains and their core proteins (proteoglycans) and glycoproteins. The distinct cellular composition and functional properties of the vascular three‐layer structure dictate its unique interstitial ECM microenvironment. ECM provides blood vessel integrity and elasticity but also is a reservoir of cytokines and growth factors, and can exert matricellular and matricrine modulation, and influence cell fate [13]. Fewer studies addressed other components of the ECM besides collagen and elastin [5] in smokers and COPD arteries, and none have studied the bronchial artery.

Therefore, we hypothesized that both pulmonary and bronchial arteries undergo significant early remodelling in smokers and patients with mild/moderate COPD. Moreover, these changes would be characterized by distinct patterns of ECM deposition and endothelial activation in the two vascular beds and the extent of these alterations would correlate with smoking history and lung function. To test this hypothesis, we performed a detailed comparative analysis of ECM components within the pulmonary and bronchial artery layers and their endothelial activation across three groups: non‐smokers, nonobstructed smokers and patients with mild/moderate COPD.

## 2 Methods

Human Research Ethics approval was obtained from São Paulo University Medical School, A.C. Camargo Hospital (São Paulo, Brazil), Leiden University Medical Centre (Leiden, The Netherlands) and Palermo University (Palermo, Italy). Informed consent was obtained from all patients prior to enrolment, which was approved by the Ethics Committee (CAPPesq 038/06). At the Leiden University Medical Center, tissue was collected according to a no‐objection system for coded anonymous further use of such tissue (www.coreon.org), and therefore individual informed consent was not required.

This study was a subanalysis of a patient's cohort undergoing lung resection surgery for primary or metastatic lung tumors from 2001 to 2007. Patients were classified into three groups, according to their (prebronchodilator) lung function: (1) non‐smokers (NS, *n* = 11): never smokers, FEV_1_ ≥ 80% predicted and FEV_1_/FVC ≥ 70%; (2) nonobstructed smokers (NOS, *n* = 14): current and/or ex‐smokers (quit ≥ 1 month) with normal lung function (FEV1 ≥ 80% predicted and FEV1/FVC ≥ 70%); and (3) COPD (*n* = 19): current and/or ex‐smokers (quitted ≥ 1 month) with COPD (FEV1 < 80% and FEV1/FVC < 70%). Detailed characteristics of the entire patient population have been described elsewhere [14].

### 2.1 Tissue Processing

One or two blocks of central airways, distant from the tumour, were collected from patients during lung resection surgery. Samples were fixed in 10% buffered formalin for 24 h, processed and paraffin‐embedded. Four‐micrometre (μm) thick sections were stained with haematoxylin and eosin (H&E) to select arteries suitable for analysis. Cases exhibiting fibrotic disorders, neoplastic tissue and poststenotic pneumonia were excluded.

Elastic fibres were identified using the Weigert's Resorcin–Fuchsin technique with oxidation [15]. Antigen retrieval methods and primary antibodies used are detailed in Table S1. Briefly, sections were dewaxed and hydrated, and endogenous peroxidase was blocked with a 0.5% hydrogen peroxide in methanol solution for 10 min at room temperature, followed by overnight incubation with the primary antibody in a humid chamber. The streptavidin‐biotin complex LSAB (Dako, Glostrup, Denmark) was used after secondary antibodies. Negative controls were performed by replacing the primary antibody with phosphate‐buffered saline and by substituting the primary antibody with an isotype‐matched antibody control.

### 2.2 Morphological Analysis

We analysed all transversely cut pulmonary and bronchial arteries available in the slides. Bronchial muscular arteries (BMA) were defined as those present in the submucosa of large airways and pulmonary muscular arteries (PMA) as those adjacent to the peripheral airways. The slides were scanned in the 3DHistech Slide Panoramic Scanner (3DHistech, Hungary) and the images analysed with the Image Pro‐Plus 4.1 for Windows software (Media Cybernetics Silver Spring, MD, USA).

Vascular structural parameters, including lumen diameter and area, tunicae intima, media and adventitia area, and vessel outer muscle perimeter (VOMP) were analysed using the modified Verhoeff–Masson trichrome staining. In BMA, the adventitial area was defined as the dense connective tissue surrounding the artery, limited by larger vessels and nerves. In PMA, the adventitial area was defined as the region extending from the outer border of the muscle layer to the alveolar attachments.

The density of elastic fibres was measured in the tunicae intima, media and adventitia. Type‐I and type‐III collagen, fibronectin, tenascin‐C, intercellular adhesion molecule (ICAM)‐1, vascular cell adhesion molecule (VCAM)‐1 and endothelin‐1 were analysed in the combined tunicae intima and media, and in tunicae adventitia, both in the pulmonary and bronchial arteries. Measurements were performed blindly to the study groups. The area of positive staining for each antibody within the marked compartments was determined by colour thresholding, as previously described [16]. Proteins' expression levels are presented as the positive area per VOMP (μm^2^/μm).

### 2.3 Statistical Analyses

Numerical data are presented as median (interquartile range [IqR]) or mean ± standard deviation [SD], according to data distribution. Depending on data distribution, one‐way ANOVA followed by a Bonferroni post hoc test or a Kruskal–Wallis test was performed to compare protein expression among the three groups: NS, NOS and COPD. We performed a full‐factorial general linear model to assess the effects of group, age and gender on the protein expression of pulmonary and bronchial vessels. The results of the general linear models are shown only for proteins' expressions that were significantly different among groups in the univariate analyses. Categorical variables were analysed using the chi‐square test. Correlations were assessed using Pearson or Spearman coefficient tests, depending on data distribution. Differences at a *p*‐value < 0.05 were considered statistically significant. Statistical analysis was performed using SPSS 22.0 software (SPSS Inc., Chicago, IL, USA).

## 3 Results

The study participants' characteristics are shown in Table 1. The NS group was significantly younger (*p* = 0.003) and had a higher proportion of females (*p* = 0.001) when compared to the NOS and COPD groups. The FEV1 and FEV1/FVC ratio were significantly different among all groups, with higher values in the NS group, followed by the NOS and COPD groups (*p* < 0.001). No statistical difference was observed between the NOS and COPD groups regarding smoking status and pack‐years.

**TABLE 1:** Demographic characteristics and lung function parameters of the study groups.

	NS (*n* = 11)	NOS (*n* = 14)	COPD (*n* = 19)	*p*
Age (year), median (IQR)	53 (43–61)	63 (58–77)	67 (60–74)	**0.003** *
Gender (*n* male)	3	10	18	**0.001** ^**‡**^
Current/ex‐smokers	0	4/10	11/8	
Pack‐years, median (IQR)	—	56 (30–80)	60 (50–80)	0.464
FEV1% pred, median (IQR)	112 (93–120)	94 (91–97)	64 (55–79)	**< 0.001** ^§^
FEV1/FVC %, median (IQR)	98 (87–103)	80 (76–92)	64 (57–68)	**< 0.001** ^**§**^

Abbreviations: COPD, chronic obstructive pulmonary disease; FEV_1_% pred, forced expiratory volume in 1 s (% predicted); FVC, forced vital capacity; IQR, interquartile range; NOS, nonobstructed smokers; NS, non‐smokers.

* Difference between NS and NOS (*p* = 0.038) and between NS and COPD (*p* = 0.002).

^‡^ Difference between NS group and NOS and COPD groups (*p* = 0.001), assessed by chi‐square test.

^§^ Difference between NS group and NOS and COPD groups (*p* < 0.001).

### 3.1 Morphometry

Morphological measurements of bronchial and pulmonary arteries across the three study groups are summarized in Table 2. A mean of 3 ± 2 bronchial (range: 1–10) and 3 ± 2 pulmonary (range: 1–9) arteries were analysed per case. The mean perimeter of bronchial arteries in NS, NOS and COPD was 0.3 mm (range: 0.1–0.8 mm), 0.4 mm (range: 0.09–1.7 mm) and 0.6 mm (range: 0.1–3.0 mm), respectively. The mean perimeter of pulmonary arteries in NS, NOS and COPD was 0.3 mm (range: 0.1–0.9 mm), 0.4 mm (range: 0.2–2.0 mm) and 0.4 mm (range: 0.2–1.0 mm), respectively. There was no difference between the three groups regarding the number of arteries analysed and blood vessel perimeters.

**TABLE 2:** Morphological structures and elastic fibre content in the bronchial and pulmonary muscular arteries.

Parameters area/VOMP (μm^2^/μm)		BMA				PMA			
		NS	NOS	COPD	P	NS	NOS	COPD	P
Morphological structures	Perimeter	0.3 (0.1–0.8)	0.4 (0.09–1.7)	0.6 (0.1–3.0)	0.14	0.3 (0.1–0.9)	0.4 (0.2–2.0)	0.4 (0.2–1.0)	0.28
	Lumen	27.14 ± 16.11	24.64 ± 9.78	31.14 ± 26.76	0.69	21.96 (17.22–39.17)	25.35 (22.35–32.32)	23.52 (19.55–34.76)	0.76
	Intimal	9.73 (4.72–27.47)	14.92 (7.11–17.79)	12.53 (8.55–27.62)	0.69	**8.88 ± 4.26**	13.23 ± 6.95	**14.67 ± 5.01**	**0.04** ^‡^
	Medial	18.85 (13.54–38.89)	22.17 (13.55–38.67)	25.75 (11.41–46.00)	0.98	18.89 ± 2.98	22.12 ± 5.20	20.37 ± 4.82	0.24
	Adventitial	**27.68 ± 11.45**	40.09 ± 9.94	**49.76 ± 23.35**	**0.009** *	42.96 ± 9.91	59.15 ± 28.72	60.44 ± 21.91	0.11
Elastic fibre	Intimal	2.75 (2.33–6.06)	9.80 (4.64–11.65)	4.18 (2.69–5.95)	0.09	**4.57 (4.00–6.68)**	**7.94 (6.83–10.77)**	8.31 (5.16–10.20)	**0.02** ^§^
	Medial	0.65 (0.32–1.65)	1.60 (1.17–2.43)	0.92 (0.23–1.81)	0.06	3.37 (1.65–4.54)	4.41 (3.06–5.96)	3.03 (0.67–5.65)	0.54
	Adventitial	3.14 (1.67–4.31)	4.54 (4.31–5.75)	3.39 (1.59–6.20)	0.06	8.05 (5.76–11.34)	9.64 (7.91–20.17)	8.36 (7.46–14.92)	0.20

*Note:* Data with a nonnormal distribution are presented as median (interquartile range) and data with a normal distribution as mean ± SD.

Abbreviations: BMA, bronchial muscular arteries; COPD, chronic obstructive pulmonary disease groups; NOS, nonobstructed smokers; NS, non‐smokers; PMA, pulmonary muscular arteries; VOMP, vessel outer muscle perimeter.

* Difference between NS and COPD groups (*p* = 0.007).

^‡^ Difference between NS and COPD groups (*p* = 0.041).

^§^ Difference between NOS and NS groups (*p* = 0.029).

**FIGURE 1:**
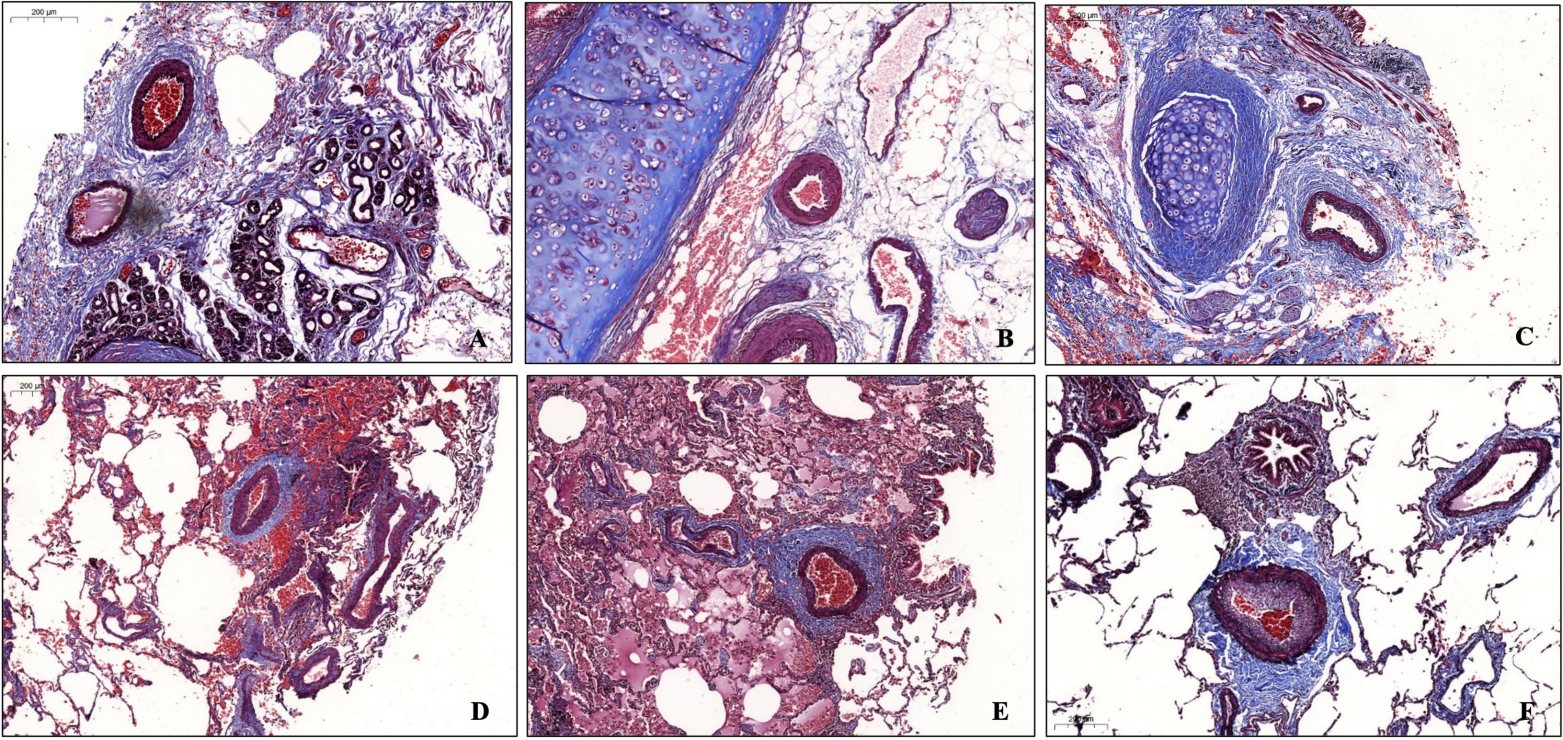
Figure depicts the morphological structures in bronchial muscular arteries (A–C) and pulmonary muscular arteries (D–F) of never smokers (A and D), nonobstructed smokers (B and E) and COPD patients (C and F). The slides were stained with Verhoeff–Masson trichrome staining. Scale bar = 200 μm.

The intimal area of PMA and adventitial area of BMA of COPD subjects was larger than NS individuals (*p* = 0.041 and *p* = 0.007, respectively), Figure 1. The PMA lumen, medial and adventitial areas had no difference between NS, NOS and COPD groups (Table 2).

### 3.2 ECM Components

Elastic fibre density in the tunicae media and adventitia of PMA was larger than BMA in the NS group (*p* < 0.001). The density of type‐I collagen in the tunicae intima/media and type‐III collagen in the tunicae adventitia of PMA was larger than BMA in the NS group (*p* = 0.001 and *p* = 0.011, respectively). The expression of tenascin‐C in the tunicae intima/media of BMA was larger than PMA in the NS group (*p* = 0.006), Table S2.

Elastic fibre protein expression within the tunicae intima, media and adventitia of BMA and PMA in the NS, NOS and COPD groups is shown in Table 2. The elastic fibre density in the PMA intimal area of NOS subjects was larger than that of NS individuals (*p* = 0.029). There was no difference in the elastic fibre content in the tunicae media and adventitia of both BMA and PMA, between the three groups (Table 2).

**FIGURE 2:**
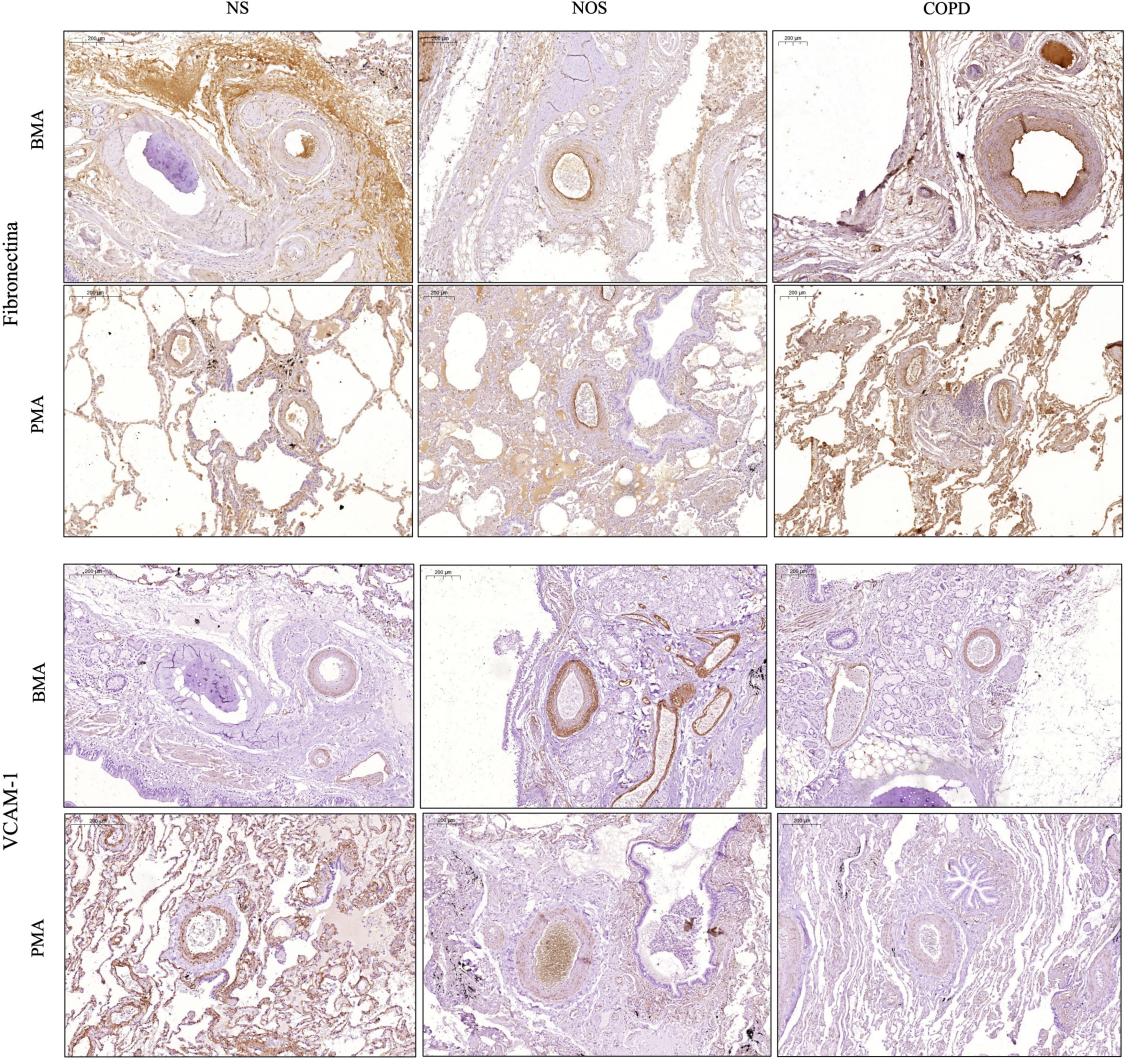
Figure depicts the fibronectin and vascular cell adhesion molecule 1 (VCAM‐1) expression in bronchial muscular arteries (BMA) and pulmonary muscular arteries (PMA) of never smokers (NS), nonobstructed smokers (NOS) and chronic obstructed pulmonary disease patients (COPD). Scale bar = 200μm.

The protein expression of type‐I and type‐III collagen, tenascin‐C and versican in the BMA and PMA did not differ among NS, NOS and COPD groups (Table 3). Similarly, the fibronectin protein expression in the tunicae intima/media and adventitia of BMA and in the tunicae adventitia of PMA was not different among the three groups (Figure 2). However, in the COPD group, the protein expression of PMA fibronectin in the tunicae intima/media was higher compared to the NS group (*p* = 0.037, Table 3).

**TABLE 3:** Type‐I and type‐III collagen, fibronectin, tenascin, vascular cell adhesion molecule (VCAM)‐1, intercellular adhesion molecule (ICAM)‐1 and endothelin‐1 (ET‐1) expression in the bronchial and pulmonary muscular arteries.

Parameters area/VOMP (μm2/μm)		BMA				PMA			
		NS	NOS	COPD	*p*	NS	NOS	COPD	*p*
Type‐I collagen	Intimal and medial	1.32 ± 0.93	1.73 ± 2.77	1.16 ± 1.35	0.79	5.25 ± 3.00	3.48 ± 2.30	4.79 ± 3.68	0.38
	Adventitial	20.51 ± 14.49	12.53 ± 7.94	12.02 ± 4.48	0.13	24.75 ± 8.24	19.05 ± 8.40	24.07 ± 8.95	0.24
Type‐III collagen	Intimal and medial	0.14 (0.08–0.44)	0.03 (0.01–0.14)	0.13 (0.01–0.26)	0.43	0.55 (0.23–1.35)	0.18 (0.13–1.46)	0.39 (0.12–0.83)	0.72
	Adventitial	5.05 ± 3.56	5.69 ± 3.86	6.07 ± 9.41	0.94	16.22 ± 10.15	16.40 ± 10.11	9.18 ± 8.67	0.12
Fibronectin	Intimal and medial	1.99 ± 1.84	2.71 ± 2.26	5.99 ± 7.25	0.27	**1.64 ± 0.85**	2.23 ± 1.42	**4.58 ± 3.22**	**0.02** *
	Adventitial	0.20 (0.03–0.38)	0.36 (0.13–2.44)	1.50 (0.41–2.74)	0.12	1.19 (0.21–1.33)	0.26 (0.04–0.84)	1.42 (0.37–1.89)	0.35
Tenascin‐C	Intimal and medial	22.42 ± 13.75	24.30 ± 17.45	30.96 ± 27.82	0.64	5.15 ± 7.08	10.27 ± 11.04	4.57 ± 4.06	0.30
	Adventitial	0.84 (0.27–1.21)	0.93 (0.42–4.00)	2.24 (0.76–10.99)	0.25	0.14 (0.09–0.75)	0.17 (0.09–2.14)	0.45 (0.20–1.07)	0.37
Versican	Intimal and medial	1.95 (0.64–6.04)	5.04 (0.84–11.50)	4.06 (1.56–6.13)	0.58	6.65 ± 7.22	9.07 ± 7.51	9.33 ± 9.47	0.69
	Adventitial	5.85 ± 3.41	9.8 ± 5.43	13.52 ± 13.25	0.12	9.33 ± 6.50	15.44 ± 10.64	14.88 ± 7.69	0.16
VCAM‐1	Intimal and medial	1.6 ± 1.10	**3.84 ± 4.28**	**1.08 ± 0.96**	**0.03** ^‡^	3.05 ± 2.56	2.97 ± 2.84	2.41 ± 2.26	0.78
	Adventitial	0.03 (0.03–0.27)	0.08 (0.01–0.29)	0.07 (0.04–0.08)	0.79	0.33 ± 0.26	0.60 ± 0.68	0.52 ± 0.59	0.50
ICAM‐1	Intimal and medial	6.99 ± 2.88	9.87 ± 8.25	8.73 ± 8.52	0.75	5.62 ± 2.60	4.83 ± 2.67	6.09 ± 2.98	0.48
	Adventitial	3.32 ± 2.05	3.71 ± 1.81	4.23 ± 3.27	0.74	3.76 ± 2.81	5.31 ± 3.77	6.29 ± 3.27	0.16
ET‐1	Intimal and medial	4.49 ± 4.33	6.61 ± 6.53	3.93 ± 2.93	0.37	2.78 ± 2.37	2.57 ± 3.34	5.69 ± 5.39	0.17
	Adventitial	0.54 ± 0.99	0.42 ± 0.55	0.54 ± 0.66	0.92	0.47 ± 0.37	0.77 ± 1.06	1.06 ± 0.69	0.25

*Note:* Data with a nonnormal distribution are presented as median (interquartile range) and data with a normal distribution as mean ± SD.

Abbreviations: BMA, bronchial muscular arteries; COPD, chronic obstructive pulmonary disease groups; NOS, nonobstructed smokers; NS, non‐smokers; PMA, pulmonary muscular arteries; VOMP, vessel outer muscle perimeter.

* Difference between COPD and NS groups (*p* = 0.037).

^‡^ Difference between COPD and NOS groups (*p* = 0.029).

In the general linear model, there was a significant effect of group on the fibronectin protein expression in the tunicae intima/media of pulmonary muscular arteries (*p* = 0.01), with age and gender adding no effect to the model.

### 3.3 Endothelial Dysfunction Markers

VCAM‐1 expression in the tunicae intima/media of BMA of the NOS group was greater than in the COPD group (*p* = 0.029) (Table 3 and Figure 2). In the general linear model, there were no significant effects of group, age and gender on VCAM‐1 expression in the tunicae intima/media of BMA.

No differences were found in the VCAM‐1 expression in the adventitial area of BMA and in any tunicae of PMA. Similarly, NS, NOS and COPD groups did not have statistically different ICAM‐1 and endothelin‐1 expression in the bronchial and pulmonary muscular arteries (Table 3).

### 3.4 Clinical‐Morphological Correlations

**FIGURE 3:**
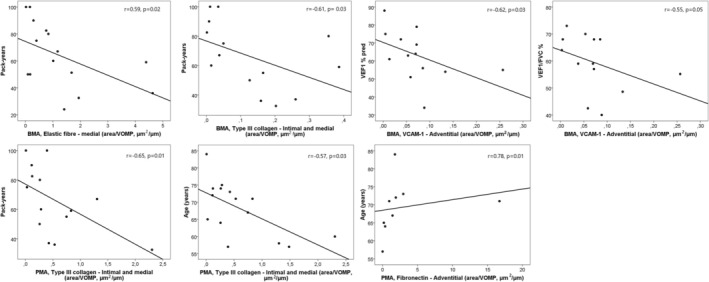
Significant correlations between extracellular matrix components and clinical variables in bronchial (BMA) and pulmonary muscular arteries (PMA).

In the COPD group, several significant correlations between ECM protein expression and clinical parameters were found. The expression of elastic fibres in the tunicae media of BMA was negatively associated with pack‐years (*r* = −0.59, *p* = 0.02). Similarly, type‐III collagen protein expressions in tunicae intima/media of BMA and PMA had a negative correlation with pack‐years (*r* = −0.61, *p* = 0.03; *r* = −0.65, *p* = 0.01, respectively) and with age (*r* = −0.57, *p* = 0.03) for PMA in the COPD group. Age was positively correlated with fibronectin expression in the tunicae adventitia of PMA (*r* = 0.78, *p* = 0.01). A negative correlation was observed between VCAM‐1 expression in the tunicae adventitia of BMA and FEV1(% predicted) (*r* = −0.62, *p* = 0.03) and FEV1/FVC (*r* = −0.55, *p* = 0.05) (Figure 3). There were no correlations with structural parameters and lung function parameters in both arteries.

## 4 Discussion

In this study we described the structural alterations, ECM remodelling and markers of endothelial activation in bronchial and pulmonary arteries of non‐smokers, smokers and patients with mild/moderate COPD. In the pulmonary arteries of COPD patients, we observed a significant thickening of the intimal layer compared to non‐smokers, accompanied by increased fibronectin deposition. In the bronchial arteries, the adventitial layer in COPD patients was thicker compared to controls. In COPD patients, elastic fibres and type‐III collagen densities were negatively correlated with pack‐years. Nonobstructed smokers had more elastic fibres in the intimal/medial layer of the pulmonary artery than controls, with increased expression of VCAM‐1 in the bronchial artery, suggesting that vascular alterations are among the early consequences of smoking.

This is the first study to address the ECM composition in the BMA in COPD. Alterations in these arteries may impact airway homeostasis, as this artery flow regulates the epithelial lining fluid and inflammatory cell influx and contracts as a response to hypoxia [10], having consequences for COPD pathogenesis. When ECM composition was compared with the pulmonary artery, bronchial arteries have less collagen and elastic fibres in their composition, but higher levels of tenascin‐C in the intimal and medial layers. Tenascin‐C is an ECM glycoprotein present in basement membranes, responsive to inflammation and linked to several processes of tissue repair [17]. It has previously been shown to be increased in the airways of patients with asthma and COPD [14, 17]. We did not find disease‐associated alterations in tenascin‐C in this study, confirming two other studies where tenascin gene and protein levels were not shown to be altered in patients with COPD [17, 18]. These results suggest that this protein may not have a significant role in vascular remodelling in this disease.

Bronchial arteries had larger adventitial areas in COPD patients, possibly as a result of peribronchial ECM deposition. Andoh et al. [19] have previously demonstrated peribronchiolar fibrosis with extension to the adjacent muscular arteries in patients with chronic bronchitis. In the bronchial microvasculature, Zanini et al. [12] detected increased expression of growth factors such as transforming growth factor beta (TGF‐β) and vascular endothelial growth factor (VEGF) in patients with untreated COPD [12], which could help explain BMA thickening.

We here show for the first time an increased expression of VCAM‐1 in the bronchial artery, but not in the pulmonary arteries of nonobstructed smokers. VCAM‐1 is an adhesion molecule known to be increased after cigarette exposure [20, 21]. In asthma, VCAM‐1 was also increased at the bronchial arteries level only [22] reinforcing the hypothesis that the mechanisms regulating leukocyte kinetics between pulmonary and bronchial circulation are different. Hogg et al. [23] suggested that, as leukocytes come in closer contact with the endothelium for prolonged periods in smaller pulmonary vessels under normal conditions, the extravasation cascade occurring in systemic vessels may be different for pulmonary vessels. Our data suggest that the bronchial circulation may be a primary site of endothelial dysfunction and inflammation in response to smoking.

Nonobstructed smokers and patients with COPD had thicker intimal layers of the PMA than non‐smoker patients, with significant changes between COPD and non‐smokers. Thickening of the intimal layer of the PMA has been previously demonstrated in some studies, being endothelial dysfunction/injury the supposed pathogenic pathway to explain this finding [5, 24]. In this study, the ECM degrading features of cigarette smoking were shown as the negative correlations of pack‐years and type‐III collagen and elastic fibres in the arteries.

Elastic fibres were increased in the intimal layer of PMA in nonobstructed smokers when compared to non‐smokers, suggesting an early, possibly maladaptive repair response. Interestingly, we found a similar pattern of elastic fibres deposition in the airways of nonobstructed smokers but not in COPD, who have fewer elastic fibres possibly due to defective repair mechanisms at this disease stage [14]. Indeed, Joglekar et al. [2] reported that patients with severe COPD have less proportional presence of latent transforming growth factor‐β binding protein 4 (LTBP4), which may result in lower TGF‐β1 activation and diminished elastogenesis. These findings could contribute to increased vascular resistance, decreased elasticity and altered tissue integrity.

Fibronectin seems to be a pivotal ECM component in the remodelling process of COPD; its gene expression has been consistently included in the gene signature of COPD [17, 25]. Fibronectin is expressed by many cells in the lung, and it is highly upregulated during tissue repair. Van Nijnatten et al. [26] recently demonstrated that, studying the COPD gene signature, fibronectin had several protein interaction networks key to the ECM remodelling, being decreased in severe COPD in relation to milder forms of the disease. Annoni et al. [14] reported increased levels of fibronectin in both small and large airways of the same group of patients with mild–moderate COPD, that were independent of smoking status or pack‐years. Here, we showed that fibronectin protein expression is increased in the PMA of mild/moderate COPD, suggesting an ongoing process of inflammation and tissue repair in the arteries that seems to be defective in the more severe cases [26].

Fibronectin has major roles in cell adhesion, proliferation and migration and regulates ECM assembly [27]. Whether the increased expression of fibronectin in PMA observed in this study represents early vascular changes that will progress to PH is unknown. Few studies analysed ECM composition in PH‐COPD. Hoffmann et al. [18] compared gene expression of remodelling genes between Pulmonary Artery Hypertension and PH‐COPD, showing no increase in collagen and tenascin genes in the COPD group. Similarly, minor changes in proteoglycans were observed in PH‐COPD when compared to other types of COPD [28]. In general, studies described more discrete remodelling alterations in PH‐COPD than in other forms of pulmonary hypertension [3].

Our study has limitations. While this study provides a crucial snapshot of early disease, a key limitation is the absence of direct measurements of pulmonary artery pressure or echocardiography data. However, it is likely that our patients did not have pulmonary hypertension, as this would have limited their eligibility for the surgery. Considering that patients with severe COPD seem to have a distinct phenotype of the disease with an increased chance of having pulmonary hypertension [29], it is probable that our findings reflect indeed early or ongoing arterial changes in COPD in mild–moderate cases. Another limitation was the younger age of the NS group, since it was composed of non‐smoking younger patients with lung metastasis such as sarcomas. Future studies are needed to determine if the changes we observed are predictive of the eventual development of pulmonary hypertension in severe COPD. This study has the strengths of being the first to perform a broad analysis of ECM components and endothelial markers at the lung tissue level and to include measurements of the bronchial artery.

In summary, nonobstructed smokers and mild/moderate COPD are characterized by ECM alterations in bronchial and pulmonary circulation, associated with arterial thickening. We show for the first time that the bronchial artery is a key site of endothelial activation in smokers and that fibronectin is a central component of pulmonary arterial remodelling in mild/moderate COPD. These findings highlight that vascular pathology is an integral and early feature of COPD.

## Author Contributions

R.A. was responsible for methodology, validation, formal analysis, writing (original draft), writing (review/editing) and visualization. S.B., N.S.X.C., M.D., P.S.H., K.F.R. and P.J.S. were responsible for writing (review/editing). J.M.B. was responsible for writing (review/editing) and data visualization. L.B.L.C. and R.C.R. were responsible for validation and formal analysis. T.M. was responsible for conceptualization, supervision, funding acquisition and writing (review/editing). All authors have approved the final version for publication.

## Funding

This work was supported by the Conselho Nacional de Desenvolvimento Científico e Tecnológico (CNPq, Brasília, Brazil) and CAPES (Coordenação de Aperfeiçoamento de Pessoal de Nível Superior, Brazil).

## Ethics Statement

Human Research Ethics approval was obtained from São Paulo University Medical School, A.C. Camargo Hospital (São Paulo, Brazil), Leiden University Medical Centre (Leiden, The Netherlands) and Palermo University (Palermo, Italy). Informed consent was obtained from all patients prior to enrolment, which was approved by the Ethics Committee (CAPPesq 038/06). Tissue in Leiden University Medical Center was collected and used according to a no‐objection system for coded anonymous further use of such tissue (www.coreon.org), and therefore, individual informed consent was not required.

## Conflicts of Interest

The authors declare no conflicts of interest.

## Supporting information


**Table S1:** Antibodies used for immunohistochemical analyses.
**Table S2:** Morphological structures, elastic fibre, type‐I and type‐III collagen, fibronectin, tenascin and versican expression in the bronchial and pulmonary muscular arteries at NS groups.

## Data Availability

The data that support the findings of this study are available from the corresponding author upon reasonable request.
