# Supplementary material for: Extracellular Matrix and Endothelial Activation Markers in the Bronchial and Pulmonary Arteries in COPD
Source: Clin Respir J. 2025 Nov 29;19(12):e70141. doi: 10.1111/crj.70141 (PMC12663766; doi:10.1111/crj.70141)
Supplement: Supplementary file 1 — Table S1: Antibodies used for immunohistochemical analyses. Table S2: Morphological structures, elastic fibre, type‐I and type‐III collagen, fibronectin, tenascin and versican expression in the bronchial and pulmonary muscular arteries at NS groups. [file CRJ-19-e70141-s001.docx]

**Supplementary material**

**Extracellular matrix and endothelial activation markers in the bronchial and pulmonary arteries in COPD**

Raquel Annoni^1,2^, Jôse Mára de Brito^1,3^, Salvatore Battaglia^4,^ Natália de Souza Xavier Costa^1^, Ligia Braga Lopes Couceiro^1^, Renata Calciolari Rossi^5^, Marisa Dolhnikoff^1^, Pieter S. Hiemstra^6^, Klaus Friedrich Rabe^7^, Peter J. Sterk^8,^ Thais Mauad^1^

**Authors’ affiliations**

1. Laboratório de Patologia Ambiental e Experimental, Departamento de Patologia, Faculdade de Medicina da Universidade de São Paulo, São Paulo, SP, Brazil.

2. Graduate Program in Rehabilitation Sciences, School of Physical Education, Physical Therapy and Occupational Therapy, Universidade Federal de Minas Gerais, Minas Gerais, Brazil.

3. Instituto Básico de Biociências, Universidade de Taubaté (UNITAU), Taubaté, SP, Brazil.

4. Division of Respiratory Diseases; Department of Promozione della Salute, Materno Infantile, Medicina Interna e Specialistica di Eccellenza 'G. D’Alessandro' – PROMISE – University of Palermo; Palermo, Italy.

5. Departamento de Patologia, Faculdade de Medicina da Universidade do Oeste Paulista, Presidente Prudente, SP.

6. Department of Pulmonology, Leiden University Medical Center, Leiden, The Netherlands

7. LungenClinic Grosshansdorf, member of the German Center for Lung Research (DZL), Germany.

8. Professor Emeritus, University of Amsterdam, Amsterdam, The Netherlands.

**Corresponding Author**

Profa. Dra. Thais Mauad, Departamento de Patologia, Faculdade de Medicina da Universidade de São Paulo, Av. Dr. Arnaldo, 455, sala 1145, Cerqueira Cesar, São Paulo, Brasil. Email: tmauad@usp.br

**Funding:** Conselho Nacional de Desenvolvimento Científico e Tecnológico (CNPq, Brasília, Brazil) and CAPES (Coordenação de Aperfeiçoamento de Pessoal de Nível Superior, Brazil).

**Table 1S:** Antibodies used for immunohistochemical analyses

| **Antibody** | **Pretreatment** | **Species** | **Dilution** | **Clone** | **Manufacturer** |
| --- | --- | --- | --- | --- | --- |
| Type-I collagen | Citrate | Goat | 1:1500 | Polyclonal | US Biological, Swampscott, MA, USA |
| Type-III collagen | Trypsin | Mouse | 1:750 | III-53 | Oncogene & Calbiochem, Darmstadt/Germany |
| Fibronectin | Citrate | Rabbit | 1:6000 | Polyclonal | Dako, Glostrup, Denmark |
| Tenascin | Pepsin | Mouse | 1:400 | BC-24 | Sigma, Saint Louis, MO/USA |
| Versican | Trypsin | Mouse | 1:1000 | 2-b-1 | Seikagaku Co., Tokyo, Japan |
| VCAM-1 | Citrate | Mouse | 1:100 | E-10 | Santa Cruz Biotechnology,  Dallas,TEXAS |
| ICAM-1 | Citrate | Mouse | 1:50 | Ncl-D54-307 | Leica Biosystems, Newcastle, UK |
| Endothelin-1 | Citrate | Goat | 1:300 | Polyclonal | Santa Cruz Biotechnology, CA, EUA |

**Table 2S:** Morphological structures, elastic fibre, type-I and –III collagen, fibronectin, tenascin and versican expression in the bronchial and pulmonary muscular arteries at NS groups.

| **Parameters - area/VOMP (µm^2^/µm)** | | **BMA** | **PMA** | **P** |
| --- | --- | --- | --- | --- |
| **Elastic fibre** | **Intimal** | 5.19±4.58 | 5.73±2.68 | 0.74 |
|  | **Medial** | 0.91±0.62 | 3.38±1.79 | **<0.001** |
|  | **Adventitial** | 3.12±1.32 | 8.16±3.10 | **<0.001** |
| **Type-I collagen** | **Intimal and medial** | 1.32±0.93 | 5.24±3.00 | **0.001** |
|  | **Adventitial** | 20.51±14.48 | 24.75±8.24 | 0.42 |
| **Type-III collagen** | **Intimal and medial** | 0.36±0.53 | 1.62±2.90 | 0.24 |
|  | **Adventitial** | 5.04±3.56 | 16.22±10.14 | **0.011** |
| **Fibronectin** | **Intimal and medial** | 1.99±1.83 | 1.63±0.85 | 0.63 |
|  | **Adventitial** | 0.66±1.23 | 0.87±0.62 | 0.68 |
| **Tenascin-C** | **Intimal and medial** | 22.42±13.74 | 5.15±7.08 | **0.006** |
|  | **Adventitial** | 1.78±2.93 | 0.49±0.66 | 0.24 |
| **Versican** | **Intimal and medial** | 4.65±7.27 | 6.64±7.21 | 0.52 |
|  | **Adventitial** | 5.84±3.40 | 9.33±6.49 | 0.13 |

**BMA:** bronchial muscular arteries; **PMA:** pulmonary muscular arteries; **VOMP:** vessel outer muscle perimeter.

Data are presented as mean±SD.
